# Setting Up a Local ADHD Service: Trials, Tribulations and Reflections on What Worked and What Went Wrong

**DOI:** 10.1192/bjo.2026.11549

**Published:** 2026-06-30

**Authors:** Dawn Collins, Lisa Riches

**Affiliations:** 1 NSFT, Great Yarmouth, United Kingdom; 2 NSFT, Norwich, United Kingdom

## Abstract

**Aims:**

To set up a comprehensive ADHD service for 18-25 years olds in our Locality, who were already under MHS

**Methods:**

Planned clinic 1 day a month, providing pre assessment sessions (45 minutes, extended screening) and full ADHD assessments (where indicated). Also offering follow ups and medication reviews for those already diagnosed. Staffing was myself (Consultant Psychiatrist) and Nursing or social work colleague.

**Results:**

The ADHD service has proved popular, busy and mostly successful: in the 1st year, we saw 21 patients and diagnosed 9 with ADHD. in 2nd year, we saw 18 patients and diagnosed 8 with ADHD. This equates to 44% of pre selected patients being diagnosed, with 56% being screened out.

Most patients opted for medication; 88% in 1st year and 100% in 2nd.

Follow ups made up the bulk of our time, with 72 hours in 2nd year, plus admin time.

Pitfalls were mostly related to time: we underestimated the sheer volume of follow ups that would be needed and the administration time (a full ADHD assessment still takes 2 -3 hours to write up comprehensively).

The Team became expert at spotting potential ADHD, making appropriate referrals to our clinic. Feedback form SU was very positive, both in terms of the process and outcomes:

***T, male 23***, *with long standing anxiety (excessive cannabis use). Not working or studying, conflict at home with Mum.*

*6 months after diagnosis (and starting medication), he has a new job (rehabilitating ex-offenders, described by him as “my dream job”), driving, stopped smoking cannabis and reports significantly reduced anxiety and that things are much better at home.*

***A, female, 21***, *not working or studying (walked out of*

*14 jobs), significant anxiety.*

*6 months after diagnosis (and starting meds),*

*volunteering with RSPCA 4 days a week (“which I love”),*

*anxiety “has gone”, driving and, still untidy but life is much*

*less chaotic and more manageable. Successfully using*

*CBT techniques and meditaiton to manager her anger.*

**Conclusion:**

The ADHD service has now become an integral part of the service we offer: we get good feedback from patients and clinicians, we are able to appropriately start and monitor medication, and we can essentially screen out almost 60% of patients. we are not, as our figures show, over diagnosing ADHD, and most patients have made excellent progress with this diagnosis and treatment.

we have reduced referrals into Adult ADHD services and have intervened at an appropriate point in young peoples lives, where managing ADHD may be critical to their well being and success (emotional, social and work wise).

